# Process evaluation of a complex, multilevel, multicomponent scheme for the prevention and control of non-communicable diseases in Tamil Nadu, India: A mixed-methods protocol

**DOI:** 10.1016/j.mex.2024.102739

**Published:** 2024-05-01

**Authors:** Manickam Ponnaiah, Joshua Chadwick, Malu Mohan, Bhavani Shankara Bagepally, Sendhilkumar Muthappan, Nandhini Prabakaran, Jerard Selvam, Harshavardhini Vasu, Viduthalai Virumbi, Aarushi Bhatnagar, Dinesh Nair, Chitra, Priya Senthil Kumar, Vidhya Viswanathan, K. Krishnaraj, V.P. Harisundari, T.S. Selvavinayagam, Darez Ahamed, S. Uma, P. Senthil Kumar, Manoj Murhekar

**Affiliations:** a ICMR – National Institute of Epidemiology, Chennai, India; b National Health Mission, Chennai, Tamil Nadu, India; c Tamil Nadu Health System Reform Program, Chennai, Tamil Nadu, India; d The World Bank, New Delhi, India; e Directorate of Medical and Rural Services, Chennai, Tamil Nadu, India; f Directorate of Medical Education, Chennai, Tamil Nadu, India; g Directorate of Public Health and Preventive Medicine, Chennai, Tamil Nadu, India; h Health and Family Welfare Department Secretariat, Chennai, Tamil Nadu, India

**Keywords:** Process evaluation, Non-communicable diseases, Door-delivery of NCD drugs, Population based screening, Makkalai thedi maruthuvam, Universal health coverage, Sustainable developmental goals, Dr Joshua and I (Manickam) have worked together on this publication and contributed equally as shared first authorship

## Abstract

**Background:**

Non-communicable diseases (NCDs) are the leading cause of morbidity and mortality in India, necessitating development of multilevel and multicomponent interventions. *Makkalai Thedi Maruthuvam* (MTM) is a complex multilevel, multicomponent intervention developed and implemented by the south Indian State of Tamil Nadu. The scheme aims to deliver services for preventing and controlling diabetes, and hypertension at doorstep. This paper describes the protocol for planning and conducting the process evaluation of the MTM scheme.

**Methods and analysis:**

The process evaluation uses mixed methods (secondary data analysis, key informant interviews, in-depth interviews, conceptual content analysis of documents, facility-based survey and non-participant observation) to evaluate the implementation of the MTM scheme. The broad evaluation questions addressed the fidelity, contexts, mechanisms of impact and challenges encountered by the scheme using the Consolidated Framework for Implementation Research (CFIR) framework. The specific evaluation questions addressed selected inputs and processes identified as critical to implementation by the stakeholders. The CFIR framework will guide the thematic analysis of the qualitative interviews to explore the adaptations and deviations introduced during implementation in various contexts. The quantitative data on the indicators developed for the specific evaluation questions will be cleaned and descriptively analysed.

## Graphical abstract

**Figure:**
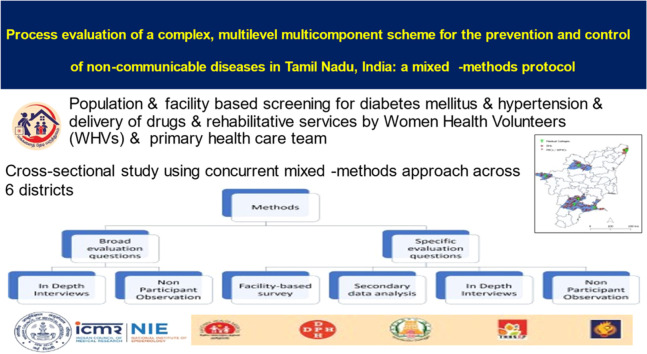

Specifications table

**Table:** 

Subject area:	Medicine and Dentistry
More specific subject area:	*Non-communicable disease*
Name of your protocol:	Process evaluation of a complex, multilevel multicomponent scheme for the management of non-communicable diseases in Tamil Nadu, India: a mixed-methods protocol
Reagents/tools:	*Not applicable*
Experimental design:	*Not applicable*
Trial registration:	*Not applicable*
Ethics:	The Institutional Human Ethics Committee of the Tamil Nadu State Directorate of Public Health Preventive Medicine has given its ethical approval on 20 May 2022 (no. DPHPM/IEC/2022/020), for this process evaluation. The interviewer will verbally confirm that participants are willing to participate in the interview and agree to have it recorded and transcribed before commencing. All evaluation-related content will be made in English or Tamil. The interviewer will ask for verbal consent and read the consent form and participant information sheet in their native language. All hard copies of interview transcripts will be stored in a secured cabinet, and all electronic data will be kept in a safe password-protected setting. We will maintain the confidentiality of the data by not using personal identifiers in the data collection form for interviews of stakeholders. Any scientific presentation or publication will not reveal the individual identity of study participants. Data will be encrypted, password-protected, and saved in the ICMR-National Institute of Epidemiology secured network once they have been processed and databases established.
Value of the Protocol:	• The World Health Organization (WHO) recommends community-based healthcare delivery models to strengthen healthcare systems in low- and middle-income countries (LMICs) and manage the increasing burden of non-communicable diseases (NCDs). However, the effectiveness of these models is yet to be evaluated. • Our study will use a mixed method approach, including the Consolidated Framework for Implementation Research (CFIR) framework, to evaluate the implemented model. • Our study will be the first to identify the implementation challenges and ways to strengthen the healthcare delivery system, which will be useful for LMIC countries to enhance the healthcare system to combat the rising prevalence of NCDs.

## Introduction

Process evaluation constitutes an extremely useful exercise in understanding how a program operates in its real-world context. A program may underperform due to several reasons, which include gaps in design, deficiencies in implementation, inadequate coverage of the target population or a combination of these. One of the key applications of process evaluation is its ability to identify the reasons for the poor performance of a program by developing a detailed, in-depth understanding of the program and evaluating how it operates in real-world contexts [1,2].

The growing recognition of the complex pathways which determine the occurrence and management of non-communicable diseases (NCDs), rooted in the eco-social context, has led to the development of multilevel and multicomponent interventions to tackle them [3,4]. The extant literature on these interventions highlights their potential to affect outcomes through a systems approach. They also reiterate the need to emphasise context-specificity, equity and integration across components [5,^6^,4].

*Makkalai Thedi Maruthuvam* (MTM) (meaning “Reaching out health services to people”) is a complex multilevel, multicomponent intervention developed and implemented in the south Indian State ‘Tamil Nadu’, by the Government of Tamil Nadu. The State, which is widely lauded for its commendable initiatives in public health, has been directing greater focus on NCDs over the past two decades [7]. The scheme, launched in August 2021, aimed to 'deliver essential healthcare at the people's doorstep.' It is being implemented by the India's National Health Mission Tamil Nadu (NHM TN), through the State Directorate of Public Health and Preventive Medicine (DPHPM), in coordination with the State Directorate of Medical and Rural Health Services (DMRHS) and that of Medical Education (DME). It offers a comprehensive range of NCD-related services for detecting, managing and preventing NCDs, including doorstep delivery of screening, oral antidiabetic and antihypertensive medications, palliative care, physiotherapy and delivery of peritoneal dialysis bags (Table 1). It also intends to meticulously add information on every line-listed beneficiary for monitoring through Tamil Nadu's Statewide platform, Population Health Registry (PHR). Trained women community health volunteers undertake the doorstep delivery of screening and medications referred to as Women Health Volunteers (WHVs). They are placed and remunerated by the State agency called Tamil Nadu Corporation for the Development of Women (TNCDW) and supervised by trained nursing professionals [named as “Mid-Level Health Providers (MLHP) and Village/Urban Health Nurses] [8,9].

**Table 1:** Types of beneficiaries eligible for home-based Makkalai Thedi Maruthuvam (MTM) services, services offered and utility of population health registry (PHR).

Type of beneficiaries for home based non-communicable diseases (NCD) drug delivery	Home-based MTM services	Utility of PHR
• Line-listed NCD patients who are 45 years andabove will be delivered hypertension/diabetesdrugs in their households	• Screening for non-communicable diseases and drug delivery to hypertension and diabetes patients	• Eligible hypertension/diabetes patients for home-based drug delivery will be linked to their respective primary care and included in the PHR at the corresponding level
• Any hypertension/diabetes patients who arehome bound or have restricted mobility due tovarious health conditions will be delivered therequired drugs to their households	• Palliative care services	• Field teams serving Block/Corporation Zone/Municipality will prioritize these registered patients for home-based services
• HT /DM Patients aged 30–44 will routinely gettheir medicines from primary care centres orfrom Mobile Medical Units as being followed inthe districts currently	• Physiotherapy services	• Patients eligible for community-based services like palliative/physiotherapy and those discharged from relevant hospital departments will also be linked to home-based services through weekly submission of eligible patient line lists to the District Deputy Health Officer (DDHS)
	• CAPD services	• The MTM Staff Nurse will ensure positive individuals are registered in the PHR, facilitating regular follow-up by the Mid-Level Health Provider (MLHP) and the Women Health Volunteer (WHV) at the community level
		• The same PHR will be used to maintain communication between the MLHP, WHV, and the Population Health Registry for effective follow-up

An intervention at the scale of MTM would greatly benefit from the insights gained from a process evaluation which has been implemented in all the districts of Tamil Nadu. Such an exploration would allow the program developers to refine the design based on the detailed description generated and provide valuable information regarding overcoming the barriers and adapting the program to suit a wide range of contexts. Hence, the Tamil Nadu Health System Reform Project (TNHSRP) and the NHM TN have collaborated with the Indian Council of Medical Research - National Institute of Epidemiology (ICMR-NIE) to undertake a process evaluation of the scheme. Based on in-depth discussions with the stakeholders, we identified that the process evaluation exercise of MTM required three major components, namely:

1. A detailed program description with its theoretical underpinnings, key components and processes.
2. Evaluation of how far the implementation has adhered to the program theory, envisaged components and processes.
3. Identify the deficiencies in inputs and processes identified in the key areas raised by the stakeholders.

In this paper, we present the steps involved in developing and finalising the protocol for the process evaluation and its conduct.

## Methods

### Positionality of the research team and reflexivity statement

The research team was diverse in their training and disciplinary and professional orientations. The core research team comprised professionals trained predominantly in biomedical or allied paradigms with advanced public health research and practice training. The rest of the research team comprised of Tamil Nadu State Health System professionals. This group was also trained predominantly in biomedical or allied paradigms and was currently engaged in administrative responsibilities in the State's public health and related cadres. Thus, public health orientation was a common thread connecting the team.

The core team was deeply trained and experienced in post-positivist research paradigms. Still, they had been exposed to varying degrees in constructivist and pragmatic paradigms through advanced training, work experience or both. The core team was heterogeneous regarding their core interest areas ranging from epidemiology, health systems and policies, and health economics. This heterogeneity, in addition to their diverse sociocultural and linguistic origins and deep experience in the cultural roots of Tamil Nadu, greatly enhanced the level of transpersonal reflexivity and critical engagement. The qualitative data collection and analysis were done by a small team of researchers (MM, JC, SM, BSB & MP) who do not belong to the state health system and are not involved in the conception or implementation of the scheme.

The team consensually problematized MTM as a complex multilevel health system intervention at the policy level. It approached the intervention multidimensionally and critically from a practical and 'problem-solving' perspective, intending to work together to refine and adapt the scheme to enhance the prevention and control of NCD in the State.

### Conceptual framework

Consequent to the growing recognition of the utility and significance of process evaluation in refining the scope and implementation of complex interventions, several frameworks have been proposed over the past two decades towards conducting and reporting them. In one of the earlier propounded frameworks, Saunders, Evans & Joshi [2] adopted a comprehensive and systematic approach to developing a process evaluation plan. The framework focused on fidelity, dose (delivered and received), reach, recruitment, and context as its key elements. A more recent framework developed by the Medical Research Council (MRC) identifies the evaluation of context, description of the intervention, implementation process, mechanisms of impact and outcomes, and the relationships between these five components as its key components [10,11].

Since the ultimate aim of any process evaluation endeavour is to refine the design and implementation and adapt them to fit better to the contexts, the exercise has to be pluralistic. It must be driven primarily by the evaluation questions on 'what works' and 'why' in real-world practice scenarios. Due to the emphasis on the consequences of the evaluation exercise rather than the methodological approach used, a pragmatic research paradigm driven by mixed methods was deemed appropriate. We combined and adapted the frameworks of Saunder, Evans and Joshi and MRC to develop a conceptual framework for this evaluation. Since the former gave a systematic and easily adaptable structure to plan and conduct a process evaluation, we merged its structure with the relevant components from both frameworks to develop this protocol.

**Fig. 1:**
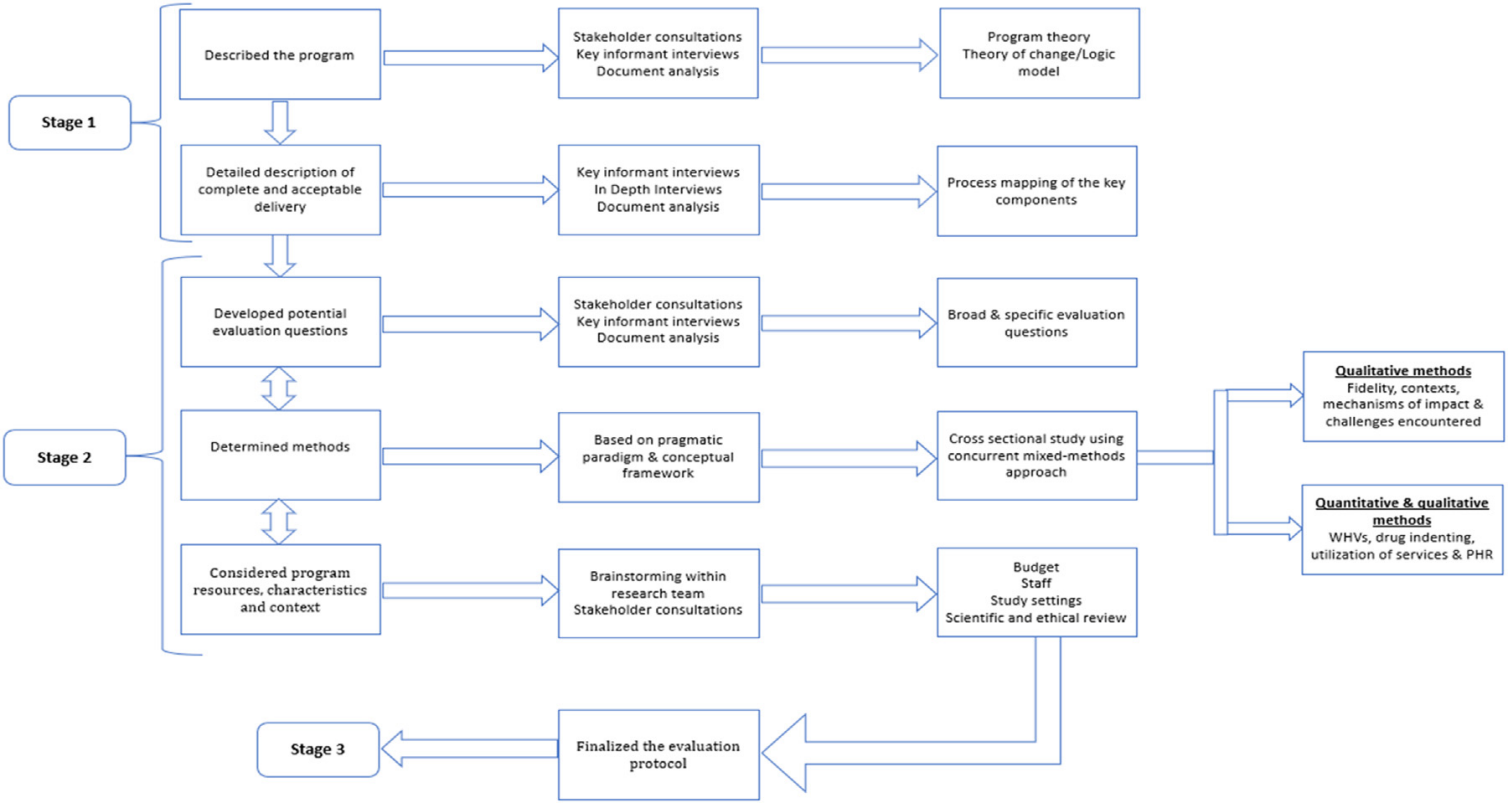
Development and finalisation of the evaluation protocol.

The key focus of the evaluation was fidelity, context, mechanisms of impact and challenges encountered during implementation. *Fidelity* was defined as the extent to which the scheme adhered to the program theory and the original design envisaged by the program developers [2]. *Context*, which has been identified as one of the most crucial determinants of the success of implementation, has been conceptualised as any factor or situation external to the program which may act as a facilitator or barrier to implementation [10]. We have conceived *mechanisms of impact* as the pathways through which the intervention was observed to lead to its outcomes as perceived by the developers, implementers and evaluators [10]. Mechanisms of impact in effect is a test of the program theory originally conceived by the program developers regarding the expected pathways through which the impact is created. The *challenges* encountered by the program implementers from various contexts were also a key component explored. The steps followed in developing the process evaluation protocol and the methods employed have been presented in Fig. 1.

### Evaluation design

This process evaluation was conducted in three primary stages:

1. Stage 1 - Description of the scheme
2. Stage 2 - Development and finalisation of the evaluation protocol
3. Stage 3 - Conduct the process evaluation

**Stage 1 - Description of the scheme**

We described the scheme in this stage, including the underlying program theory, overall goal, purpose, and specific objectives. The key components of the scheme and their interrelationships, processes within each of the components and the major inputs required to operate the scheme were also captured. This entire stage was accomplished in two steps:

In the *first step*, we developed the program theory and logic model for the program through stakeholder consultations and conceptual content analysis of key scheme-related documents (Supplementary file 1). The MTM scheme was introduced statewide to conduct population-based screening for diabetes and hypertension among individuals aged 18 and above. The initiative also involves the delivery of hypertension and diabetes drugs to registered patients aged 45 and above, as well as those with restricted or poor mobility.

As of March 2023, the departmental data reveals that a total of 1,0 046,429 patients have received initial services, and an additional 27,690,102 patients have benefited from repeat services.

In the *second step*, we mapped the processes in minute detail in each of the five key components identified - population-based screening, facility-based screening, doorstep delivery of drugs, doorstep delivery of rehabilitative services and overall monitoring and supervision of the scheme. This step was achieved through key informant interviews with the program implementers from NHM and DPH, in-depth interviews and documentary analysis. In addition to the program developers, we interviewed DMRHS, DME, TNHSRP and TNCDW stakeholders in multiple rounds. We also interviewed some District Program Officers who acted as nodal officers of MTM and the Continuous Ambulatory Peritoneal Dialysis (CAPD) component of MTM and PHR.

By the end of the stage, we developed a logic model of the scheme down to the specific objectives, key processes and inputs and drew detailed maps of the key processes in each of the identified components of the scheme.

**Stage 2 - Development and finalisation of the evaluation protocol**

The protocol for the process evaluation was developed and finalised in the following three key steps, which were iterative and non-linear. That is, at each of these three steps, the research team could return to either of the other two steps and explore further to refine the protocol.

***1. Development of the list of evaluation questions***

In this step, we presented our detailed program description to each stakeholder in separate and group meetings to refine it further. Subsequently, after multiple iterations, we reached a consensus regarding the program description and broad evaluation questions presented below.

(i) To what extent is the implementation of the MTM scheme adhering to its proposed design?
(ii) What are the contextual barriers and facilitators to implementing MTM according to its proposed design?
(iii) What are the challenges encountered in implementing MTM from the perspectives of the stakeholders?

However, the stakeholders also suggested four critical areas of specific exploration on the basis on which the following specific evaluation questions were framed:

(i) What is the current status of the key field-level volunteers of the scheme engaged in population-based screening and doorstep delivery of drugs, namely the Women Health Volunteers (WHVs), regarding their placement, training, knowledge and confidence?
(ii) How has the home-based delivery of MTM services been reflected in the pattern of indenting of Oral Antidiabetic Drugs (OAD) & antihypertensive medications?
(iii) How has the home-based delivery of drugs been reflected in the utilisation pattern of health facilities among patients with diabetes mellitus and hypertension?
(iv) What is the status of the linkage of the information system under MTM with the State Population Health Registry (PHR)?

***2. Determination of evaluation methods***

The evaluation methods were guided by the conceptual framework, which shaped the broad evaluation questions, the requirements of the specific evaluation questions and the research paradigm, all three of which were agreed upon by the research team and the key stakeholders. It was decided that the broad evaluation questions would be explored using qualitative methods and the specific evaluation questions employing mixed methods (Table 3).

**Table 2:** Study settings selected for the process evaluation and rationale for selection.

District selected	Rationale for selection	Estimated population (2023)
Chennai	Urban district with a high performer in Social Progress Index* (74.92)	11,933,000
Madurai	Predominantly urban district with a significant proportion of the rural population (based on 2011 Census) and a medium performer in Social Progress Index (64.68)	1766,000
Viruthunagar	Predominantly rural district (based on 2011 Census) and a medium performer in Social Progress Index (61.16). One of the two districts selected for the Aspirational Districts Program by Niti Aayog.	962,062
The Nilgiris	Hilly district with difficult and hard to access terrain and a medium performer in Social Progress Index (64.68)	970,721
Ramanathapuram	Predominantly rural district with a high proportion of Scheduled Caste population (based on 2011 Census) and a low performer in Social Progress Index (59.62). One of the two districts selected for the Aspirational Districts Program by Niti Aayog.	1510,000
Dharmapuri	Predominantly rural district with a significant proportion of Scheduled castes and Scheduled tribes (based on 2011 Census) and low performer in Social Progress Index (58.99)	1245,931

⁎ Social Progress Index (SPI) is based on the assumption that economic measures alone do not capture social progress and specifically track social outcomes which include access to schools, healthcare, sanitation and nutrition at national and sub-national levels. The index is developed based on three broad dimensions of datasets: basic human needs, foundations of wellbeing and opportunity.

**Table 3:** Program matrix to evaluate the specific evaluation questions.

Areas addressed	Programmatic level	Data required	Quantitative methods	Data required	Qualitative methods
Women Health Volunteers	Inputs	Recruitment Attendance Incentivization Training	Facility-based survey	Barriers to recruitment of WHVs Reasons for their poor retention Perceptions of WHVs about: • Nature of work • Regularity & adequacyof incentives • Adequacy of trainingExperiences of WHVsregarding: • Difficulties encounteredin work • Positive and negativeexperiences at workConfidence indelivering the tasks	In Depth Interviews with WHVs & other facility-based stakeholders Non-Participant Observation of WHVs’ activities
	Processes	Review meetings			
	Outputs	Knowledge Achievement of screening & drug delivery targets			
Patterns of drug indenting	Inputs	Difference in the proportions of indenting of Oral Antidiabetic Drugs (OAD) & antihypertensive medications at government health facilities	Secondary data analysis - facility level data on the drug indenting between January and May in 2018 (before MTM) and January and May in 2022 (after MTM)	Perceptions regarding the patterns of drug indenting and procurement at various health facilities consequent to the implementation of MTM	In Depth Interviews with district level and facility level stakeholders
Patterns of utilization of government health facilities	Inputs	Difference in the proportions of patients utilizing government health facilities for diabetes and/or hypertension care	Secondary data analysis - facility level data on the utilization of government health facilities between January and May in 2018 (before MTM) and January and May in 2022 (after MTM)	Perceptions regarding the utilization of various health facilities for diabetes and/or hypertension care consequent to the implementation of MTM Challenges in streamlining patients from secondary and tertiary level health facilities to primary health level facilities	In Depth Interviews with district level and facility level stakeholders
				Perceptions and experiences of current beneficiaries of MTM.	Beneficiaries of the scheme who: • Exclusively usegovernment healthfacilities for routinecare • Partially or completelyuse private healthfacilities for routinecare • Experiences with thescheme
Population Health Registry	Inputs	Training for PHR linking	Facility-based survey	Challenges faced in regular linkage of MTM beneficiary list to PHR	In Depth Interviews with WHVs & other facility-based stakeholders Non-Participant Observation of WHVs’ activities
	Processes	PHR linking			

***3. Consideration of program resources, characteristics and context***

Once the methods were determined, we engaged deeply with the two key stakeholders, namely TNHSRP and TN—NHM, towards finalising the protocol, including the study settings, research team, recruitment of field staff to be engaged in the quantitative data collection, the budget and, establishing the anticipated duration for the evaluation—set at six months. The protocol was finalised after reaching a consensus through multiple rounds of meetings. It was reviewed and approved by the technical and ethics review committees of DPHPM (DPHPM/IEC/2022/020). The data collection commenced in June 2022.

**Stage 3 - Conducting the process evaluation**

Concurrent explanatory mixed methods approach using a cross-sectional design was employed.

### Evaluation settings

Six districts across the State of Tamil Nadu were selected to ensure that the settings represented the diversity of the State in terms of the socio-demographic composition of the population, physical accessibility and Social Progress Index (SPI) (Table 1). We selected Chennai, Dharmapuri, Madurai, Ramanathapuram, The Nilgiris and Virudhunagar as study districts. The evaluation was conducted in primary health centres (PHCs) and Sectors/Health Sub centres (HSCs) from urban and rural settings in the study districts.

### Addressing the broad evaluation questions

The broad evaluation questions, which focused on fidelity, context, mechanisms of impact and challenges encountered, were explored using qualitative methods. The exploration was guided by the *Consolidated Framework for Implementation Research (CFIR)*, a metatheoretical framework that promotes enquiries into ‘what works’ and ‘why does it work’ [12].

Based on this, we explored four key constructs of implementation within MTM: (i) the ‘core’ intervention characteristics, which are essential and unavoidable to maintain its theoretical integrity and the adaptable peripheral characteristics, which can be modified without affecting the integrity (ii) the context, characterised by the outer and inner settings; the outer setting being the larger socio-economic and political context within which the health system resides, and the inner setting being the system's structural, political and cultural milieu, (iii) the individuals or the stakeholders who developed, implemented and benefitted from the scheme, their perceptions and experiences regarding the mechanisms of impact and challenges to implementation, and (iv) the extent to which the implementation processes adhere to the program theory and description in real-world contexts and deviate from them. We explored three aspects, namely, what works, what does not work and why, and how the status of implementation affects the integrity of the program theory. This exploration was achieved by contextualising the process maps developed in the description stage to identify the *adaptations* (changes made during implementation which do not affect the integrity of the program theory) and *deviations* (changes made which affect the integrity of the program theory) in each of the selected study settings.

• *Sources of data*: Program implementing stakeholders, implementation of the scheme occurring in selected settings and PHC and HSC based scheme-related records.
• *Study methods:* In-depth interviews with program implementing stakeholders at three levels of administration, semi-structured non-participant observation of scheme implementation - screening, monitoring and doorstep delivery of medications and other services at the facilities and community levels and validation of the findings using the facility-based scheme-related records (Fig. 3).

**Fig. 2:**
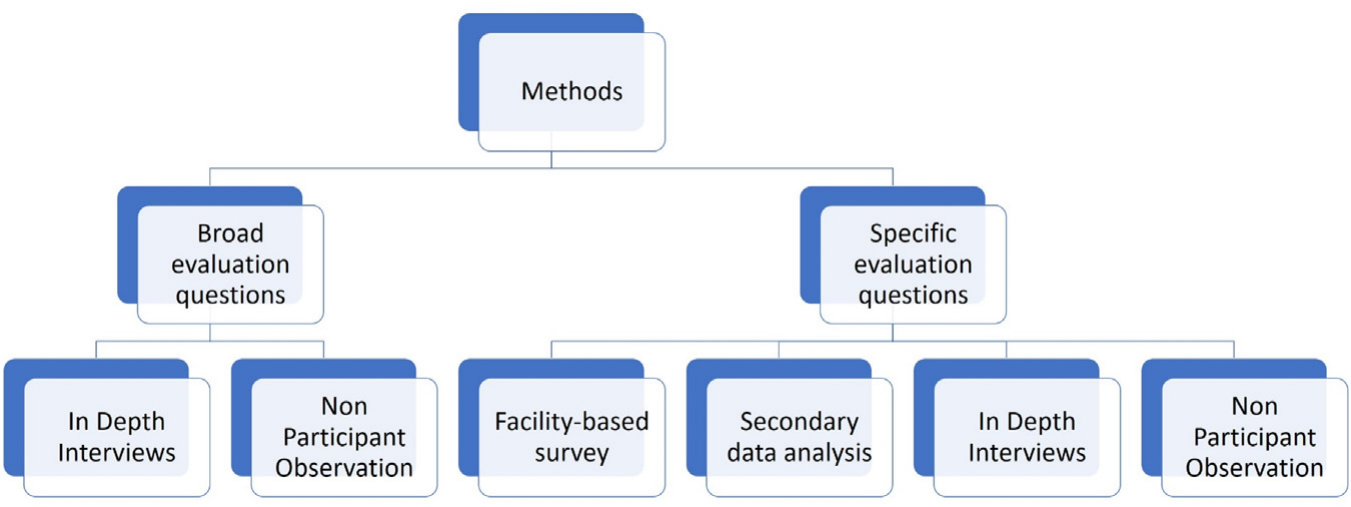
Methods used to address the broad and specific evaluation questions.

**Fig. 3:**
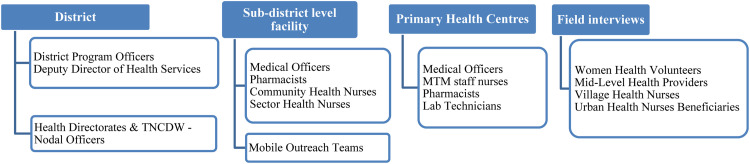
Stakeholders interviewed to address the evaluation questions.
• *Study participants:* At the first level, we interviewed the program-implementing stakeholders at the district level. The facility-level implementing officials from urban and rural health facilities were interviewed at the second level. The facilities at this level were identified first through a *pile-sorting exercise* with the district-level stakeholders. The PHCs which stood out in terms of performance (high and low) in each of the six districts were selected. At the third level, we interviewed the WHVs, the primary workforce delivering the scheme in the community and other field-level health workers (Fig. 2). The key informants from the state level directorates and offices were regularly consulted for any additional information or clarification regarding the influence of the larger context on the scheme implementation in specific study settings.
• *Sampling:* The study participants were selected purposively using criteria sampling. The stakeholders’ role in the implementation of the scheme at each administrative level (district, facility and field), and the representation of both rural and urban PHCs in each of the study districts determined their selection. The qualitative data analysis occurred simultaneously with the data collection and hence, the number of interviews in each participant category, within each district, was determined by the information saturation.

### Addressing the specific evaluation questions

We developed matrices for each of the four components addressed in the specific evaluation questions to decide which methods (qualitative/quantitative/both) are required (Table 2). After identifying the appropriate methods based on the data required, the indicators to evaluate the quantitative data were developed (Supplementary file 2. Subsequently, appropriate tools were developed and pilot-tested, and the field investigators recruited for the facility-based survey were trained.

**Fig. 4:**
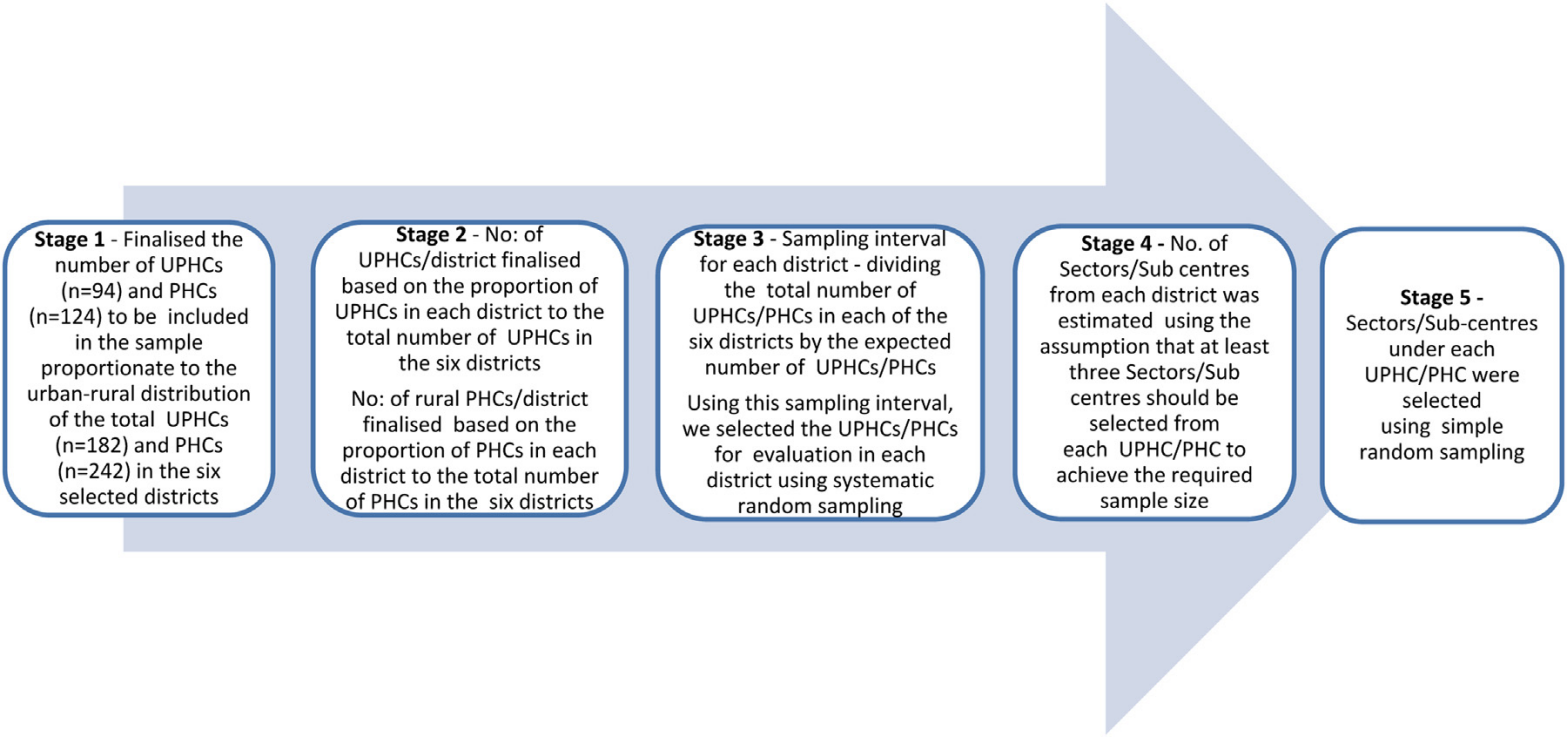
Sampling for the facility-based survey.
  For the qualitative component, in addition to the program implementing stakeholders interviewed to address the broad evaluation questions, we also interviewed the scheme beneficiaries. They were selected purposively using maximum variability sampling to ensure representation of diverse class, caste and gender identities relevant to each study context.
• *Sample size determination for the facility-based survey:* We included 660 HSCs (=WHVs) for the assumptions of regular services by the WHVs for the past two months in the HSCs as 50 %, with 5 % absolute precision, 95 % confidence interval, and a design effect of 2.0 (PHC as a cluster). The sample size of 660 WHVs = 220 PHCs * 3 HSCs, i.e., 3 HSCs from each of the selected PHCs.

• *Sources of data:* The data sources considered are program implementing stakeholders at district, facility and field levels; implementation of the scheme at facilities and field; records from surveyed facilities including MTM registers, attendance records of WHVs, fixed tour plans of the field teams and line lists of MTM beneficiaries. Facility-based data on drug indenting by and utilisation of government health facilities by the scheme beneficiaries, compiled from all the districts, was also used.
• *Study methods:* The quantitative methods are interviews as part of the facility-based survey and secondary data analysis of compiled facility-based data on drug indenting and utilisation. The qualitative methods employed are in-depth interviews and semi-structured non-participant observation. Non-participant observation was conducted to explore the confidence levels of the WHVs in delivering their tasks and to get a direct understanding of the day-to-day challenges faced by the scheme. Detailed in-depth interview guides and a semi-structured observation checklists are developed for each study participant category.
• *Study participants:* The study participants for the facility-based survey included Women Health Volunteers, Medical Officers, MTM Staff Nurses, Village/Urban Health Nurses and Mid-Level Health Providers. In-depth, interviews were conducted among the program-implementing stakeholders at district and facility levels. Two categories of beneficiaries were selected using simple random sampling from two separate line lists of the WHVs who were interviewed:
  ○ Line list of those screened at the doorstep over the two months preceding the evaluation
  ○ Line list of eligible beneficiaries who were delivered drugs at the doorstep over the four months preceding the evaluation
  ○ One beneficiary was selected from the line lists of all the WHVs interviewed as part of the evaluation.
• *Sampling:* For the facility-based survey, primary (UPHCs, PHCs, Sectors, HSCs), secondary and tertiary government health facilities were selected through appropriate random sampling procedures. There were 424 PHCs, with 242 in rural and 182 in urban areas in the six selected districts. In total, 2146 HSCs were there (each HSC will have a WHV), with 931 urban HSCs and 1215 rural HSCs in the six selected districts. The sampling of UPHCs/PHCs and Sectors/HSCs was done in five stages (Fig. 4). The secondary and tertiary health facilities were selected from the selected districts using simple random sampling, ensuring that at least two secondary and one tertiary facility were selected from each of the six districts.

### Plan for analysis

The qualitative interviews, scheme-related documents and field notes were transcribed and deductively coded, guided by the CFIR framework and thematically analysed to answer the broad and specific evaluation questions. To answer the broad evaluation question related to fidelity of implementation, we did a comparative analysis of the process maps developed during the description of the scheme with that developed during the evaluation from every study setting to document the key adaptations and deviations introduced. The contextual factors necessitating these adaptations and deviations and how the deviations affect the mechanisms of impact and the integrity of the program theory were also documented.

The quantitative data pertaining to the indicators developed for the specific evaluation questions is cleaned and descriptively analysed. The indicators primarily focus on the inputs and processes necessary to ensure the smooth implementation of the scheme, and hence the indicators will shed light on the deficiencies in this regard. The qualitative data collected towards the specific evaluation questions is useful to explain many of the indicators. For instance, the barriers to recruiting WHVs or retaining them at work, as perceived by the stakeholders, explain the deficiencies in their placement and poor retention. The qualitative data also offers greater nuances to certain indicators. For instance, the perceptions of WHVs on the adequacy of their training may offer deeper insights than the mere proportion of WHVs who received training.

These insights from the broad evaluation questions will be shared with the program developing and implementing stakeholders, and the entire team will revisit the scheme's design. The focus of this exercise will be to incorporate the contextual adaptations which either enhance or at least preserve the integrity of the original intervention into the program design. The revised design will also avoid the potential for deviations by addressing the contextual factors necessitating them. The answers to the specific evaluation questions will guide the stakeholders to address the deficiencies in inputs and processes about specific components. It will also give crucial insights into what aspects are being implemented well.

### Quality assurance and quality control mechanisms

Quality assurance of the evaluation was achieved through thorough and multiple reviews of the protocol, use of conceptual and analytical frameworks in protocol development, thorough training of data collectors, pilot testing and careful, culturally nuanced Tamil translated tools. Daily review meetings during data collection, regular review of data collected, use of checklists to track all the components of data collection, supportive supervision to the field assistants and random cross-checking of the data are used for quality control.

## Discussion

In this paper, we have presented a detailed protocol of the process evaluation of a complex intervention that we undertook across six districts of the Indian State of Tamil Nadu. According to the MRC, the complexity of an intervention is determined by the difficulty experienced in defining its 'active ingredients' and their interrelationship [13]. The assessment of such complexity is possible only when an intervention is described with adequate detail, and hence, expectedly, that constitutes the first step.

Multilevel interventions act on multiple organisational levels and diverse contexts, while a multicomponent intervention uses more than one strategy or mechanism to achieve its objectives [3,4]. As a statewide intervention operating at district, sub-district, facility, community and individual levels, with a combination of strategies, MTM qualifies as a multilevel and multicomponent intervention. There are several challenges in implementing such a scheme in a low- and middle-income setting like India, with considerable resource constraints, primarily the integration across contexts, levels and strategies.

An evaluation protocol to study the implementation of such a scheme should be broad enough to capture its scale in terms of all its components and a range of contexts. However, it should also be specific enough to capture the processes involved in detail so that implementation fidelity can be properly evaluated. According to the updated MRC guidance, a trade-off exists between precise, unbiased answers to specific questions and more uncertain answers to broader questions. The document suggests that the research team should choose the questions which provide the most useful answers to the stakeholders, even at the cost of relatively lower certainty, rather than those which provide more precise answers [11]. In our study, we adopted a broader approach to evaluating the scheme's implementation and reserved a narrower approach to questions that the stakeholders raised. The eventual utility of the findings of the exercise to the stakeholders was the most important consideration guiding the research team. This intent also guided the use of a pragmatic framework and mixed methods for evaluation.

Closely engaging the health system stakeholders, thereby closing the gap between knowledge production through research and its use, has been a key challenge to health systems research [14,15]. One of the major highlights of this evaluation has been the close association between the core research team and the stakeholders at all program development and implementation levels. Such coproduction of knowledge enhances the usability of the data produced and the chances of the data being used to refine the program. The potential of interventions interacting with their contexts to affect changes and the significance of stakeholder perceptions have been highlighted in the update on MRC guidelines. Our protocol has duly acknowledged the significance of both these factors for program implementation and has been developed accordingly.

There are several practical frameworks guiding the development of evaluation plans. But published protocols based on evaluating specific health programs, which can comprehensively guide a newly constituted process evaluation team, are limited. We have extensively relied on the process evaluation plan by Saunders, Evans & Joshi (2005) and the guidance documents from MRC (2015 & 2021) in developing this protocol. Despite the iterative nature of the protocol development process, we managed to capture the key steps involved, enhancing potential replicability and utility of the protocol for other health researchers. Moreover, there are some limitations to this study. This study included the participants who are line listed as beneficiaries of the program, but not all the eligible beneficiaries. Secondly, we did not sample the study settings due to resource constraint. However, we ensured the study settings to be geographically representative in consultation with the State program implementers.

## Declaration of competing interest

The authors declare that they have no known competing financial interests or personal relationships that could have appeared to influence the work reported in this paper.

## Data Availability

No data was used for the research described in the article.

## Appendix Supplementary materials

Image, application 1
