# Supplementary material for: Process evaluation of a complex, multilevel, multicomponent scheme for the prevention and control of non-communicable diseases in Tamil Nadu, India: A mixed-methods protocol
Source: MethodsX. 2024 May 1;12:102739. doi: 10.1016/j.mex.2024.102739 (PMC11087990; doi:10.1016/j.mex.2024.102739)
Supplement: Supplementary file 1 [file mmc1.docx]

**Annex S1. Program matrix to evaluate the Makkalai Thedi Maruthuvam (MTM) ‘to deliver essential healthcare to the doorstep of the people’ scheme, Tamil Nadu, India, 2022**

**Table 1. Program matrix to evaluate the current status and functioning of the WHVs in MTM**

| **Level** | **Program element** | **Quantitative methods** | | | | **Qualitative methods** | |
| --- | --- | --- | --- | --- | --- | --- | --- |
|  | | Indicators | Data needed | Data collection method | | Data needed | Data collection method |
| Input  (1.A) | 1.A.1 | Proportion of Health Sub Centres (HSCs) with a vacant post for WHVs at the time of the study | Numerator: Number of HSCs with vacant posts at the time of the study | | Verified from the state list of total WHVs  Cross-sectional study of HSCs – Village Health Nurse at the HSCs, MTM nurse/MO at the respective PHC  Review of attendance register of WHVs | - Potential reasons for poor retention of WHVs - Challenges faced by the WHVs in regular reporting | A. In Depth Interviews of WHVs |
|  |  |  | Denominator: Total number of HSCs surveyed | |  |  |  |
|  | 1.A.2 | Proportion of WHVs who have been regularly reporting to a Health Sub Centre for the past eight weeks | Numerator: Number of WHVs who have been regularly reporting to a HSC for the past eight weeks | | Cross sectional study of HSCs – Village Health Nurse at the HSCs, MTM nurse/MO at the respective PHC |  |  |
|  |  |  | Denominator: Total number of WHVs who participated in the study | |  |  |  |
|  | 1.A.3 | Proportion of WHVs who have regularly received their monthly incentive | Numerator: Number of WHVs who have regularly received their monthly incentive | | Cross-sectional study of WHVs | - Perceptions of WHVs regarding the regularity and mode of remuneration and incentivization | In Depth Interviews with WHVs |
|  |  |  | Denominator: Total number of WHVs who participated in the study | |  |  |  |
|  | 1.A.4 | Proportion of WHVs who have regularly received their performance incentive | Numerator: Number of WHVs who have regularly received their performance incentive | | Cross-sectional study of WHVs |  |  |
|  |  |  | Denominator: Total number of WHVs who participated in the study | |  |  |  |
| Process  (1.B) | 1.B.1 | Proportion of WHVs who have received training to deliver MTM services | Numerator: Number of WHVs have received training* to deliver MTM services | | Cross-sectional study of WHVs | - Challenges faced by the WHVs due to the lack of training or due to inadequate training - Satisfaction with the training received by the WHVs | A. In Depth Interviews with WHVs who have received training and those who have not received training  B. Ethnographic observation of the interphase between WHVs and beneficiaries  C. In Depth Interviews with facility-based stakeholders (Medical Officers, MTM nurses, Mid-Level Health Providers and/or Village Health Nurses) |
|  |  |  | Denominator: Total number of WHVs who participated in the study | |  |  |  |
|  | 1.B.2 | Proportion of WHVs who have attended all the Tuesday review meetings at the PHC over the past four weeks | Numerator: Number of WHVs who have attended all the Tuesday review meetings at the PHC over the past four weeks | | Cross-sectional study of WHVs |  |  |
|  |  |  | Denominator: Total number of WHVs who participated in the study | |  |  |  |
| Output  (1.C) | 1.C.1 | Proportion of WHVs who have correct knowledge of the screening cut off for diabetes and hypertension | Numerator: Number of WHVs who have correct knowledge of the screening cut off for diabetes and hypertension | | Cross-sectional study of WHVs | - Satisfaction of WHVs with the quality of inventory provided - Perceptions of facility-based stakeholders regarding the quality of inventory provided to WHVs | A. Ethnographic observation of the interphase between WHVs and beneficiaries  B. In Depth Interviews with WHVs, facility-based stakeholders (Medical Officers, MTM nurses, Mid-Level Health Providers and/or Village Health Nurses) |
|  |  |  | Denominator: Total number of WHVs who participated in the study | |  |  |  |
|  | 1.C.2 | Proportion of WHVs who have correct knowledge of the referral algorithm for home-based monitoring of diabetes mellitus and hypertension under MTM | Numerator: Number of WHVs who have correct knowledge of the referral algorithm under MTM | | Cross-sectional study of WHVs |  |  |
|  |  |  | Denominator: Total number of WHVs who participated in the study | |  |  |  |
|  | 1.C.3 | Proportion of WHVs who completed their daily screening target at least seven days over the past four weeks of screening | Numerator: Number of WHVs who completed their daily screening target uat least seven days over the past four weeks of screening | | Cross-sectional study of WHVs  Review of WHV work logbook of screened individuals  Review of Master Register for PBS at the PHC | - Challenges faced in achieving targets | A. Ethnographic observation of WHVs on a regular work day  B. In Depth Interviews with WHVs  B. In Depth Interviews with facility-based stakeholders (Medical Officers, MTM nurses, Mid-Level Health Providers and/or Village Health Nurses) |
|  |  |  | Denominator: Total number of WHVs who participated in the study | |  |  |  |
|  | 1.C.4 | Proportion of individuals referred to PHC by the WHV over the past eight weeks | Numerator: Number of individuals referred to PHC over the past eight weeks | | Review of WHV work logbook of screened individuals  Telephonic interviews of screened individuals | - Perceptions of community members regarding MTM scheme - Perceptions regarding the performance and behaviour of WHVs - Reasons for loss in screening cascade among those screened to post screening referral to PHC | A. Ethnographic observation of WHVs one a regular work day  B. In Depth Interviews with community members  C. In Depth Interviews with the individuals screened by the WHVs |
|  |  |  | Denominator: Total number screened over the past eight weeks | |  |  |  |
|  | 1.C.5 | Proportion of individuals who successfully completed their post-screening referral over the past eight weeks | Numerator: Number of individuals who successfully completed their post-screening referral over the past eight weeks | | Review of WHV work logbook of screened individuals  Telephonic interviews of referred individuals |  |  |
|  |  |  | Denominator: Total number of individuals referred to PHCs after screening over the past eight weeks | |  |  |  |
|  | 1.C.6 | Proportion of individuals newly screened to have systemic hypertension (SHTN) and/or diabetes mellitus (DM) by the WHVs over the past eight weeks | Numerator: Number of individuals newly screened to have systemic hypertension (SHTN) and/or diabetes mellitus (DM) by the WHVs over the past eight weeks | | Review of WHV’s line list of MTM beneficiaries  Review of family folders  Telephonic interviews of referred individuals  Review of MTM treatment cards (whenever possible)  Review of MTM register at PHC | Perceptions of current beneficiaries of home-delivery of drugs regarding:   1. MTM scheme 2. Performance and behavior of WHVs 3. Trust in WHVs 4. Drug delivery and referral protocol | A. In Depth Interviews with beneficiaries of home delivery of drugs under MTM |
|  |  |  | Denominator: Total number screened by the WHV over the past eight weeks | |  |  |  |
|  | 1.C.7 | Proportion of individuals who were newly diagnosed at PHC to have systemic hypertension (SHTN) and/or diabetes mellitus (DM) among those screened and referred by the WHVs over the past eight weeks | Numerator: Number of patients who were newly diagnosed at PHC to have systemic hypertension (SHTN) and/or diabetes mellitus (DM) over the past eight weeks | | Review of WHV’s line list of MTM beneficiaries  Review of family folders  Telephonic interviews of referred individuals  Review of MTM treatment cards (whenever possible)  Review of MTM register at PHC |  |  |
|  |  |  | Denominator: Total number of individuals screened and referred by the WHVs to the PHC over the past eight weeks for systemic hypertension (SHTN) and/or diabetes mellitus (DM) | |  |  |  |
|  | 1.C.8 | Proportion of the newly diagnosed patients over the past eight weeks who are currently provided home-based delivery of drugs by the WHV | Numerator: Number of the newly diagnosed patients over the past eight weeks who are currently provided home-based delivery of drugs by the WHV | | Review of WHV’s line list of MTM beneficiaries  Review of family folders  Telephonic interviews of referred individuals  Review of MTM treatment cards (whenever possible)  Review of MTM register at PHC |  |  |
|  |  |  | Total number of individuals screened and referred by the WHVs to the PHC over the past eight weeks for systemic hypertension (SHTN) and/or diabetes mellitus (DM) | |  |  |  |
|  | 1.C.9 | Proportion of current beneficiaries whose blood pressure and/or blood glucose are under control, who were referred by the WHV for the routine follow up review over the past eight weeks | Numerator: Number of current beneficiaries whose blood pressure and/or blood glucose are under control, who were referred by the WHV for the routine follow up review over the past eight weeks | | Review of WHV’s line list of MTM beneficiaries  Review of family folders  Telephonic interviews of referred beneficiaries  Review of MTM treatment cards (whenever possible)  Review of MTM register at PHC |  |  |
|  |  |  | Denominator: Total number of current beneficiaries visited by the WHV over the past eight weeks who were due for their routine follow up review (BP/sugar under control and medication taken for two months) | |  |  |  |
|  | 1.C.10 | Proportion of current beneficiaries whose blood pressure and/or blood glucose are not under control, who were referred by the WHV for review over the past eight weeks | Numerator: Number of current beneficiaries whose blood pressure and/or blood glucose are not under control, who were referred by the WHV for review over the past eight weeks | | Review of WHV’s line list of MTM beneficiaries  Telephonic interviews of referred beneficiaries  Review of MTM treatment cards (whenever possible)  Review of MTM register at PHC |  |  |
|  |  |  | Denominator: Total number of current beneficiaries visited by the WHV over the past eight weeks who were due for a non-routine review (BP/DM not under control) | |  |  |  |
|  | 1.C.11 | Proportion of current beneficiaries whose blood pressure and/or blood glucose are under control, who have successfully completed their follow up review at the time of the study | Numerator: Number of current beneficiaries whose blood pressure and/or blood glucose are under control, who have successfully completed their follow up review at the time of the study | | Review of WHV’s line list of MTM beneficiaries  Telephonic interviews of referred beneficiaries  Review of MTM treatment cards (whenever possible)  Review of MTM register at PHC | Perceptions regarding referral reviews at facilities and reasons for poor/delayed adherence | In Depth Interviews with beneficiaries of home delivery of drugs under MTM who have not completed their referral reviews at facilities  In Depth Interviews with WHVs |
|  |  |  | Denominator: Total number of beneficiaries referred by the WHV for their routine follow-up review over the past eight weeks | |  |  |  |
|  | 1.C.12 | Proportion of current beneficiaries whose blood pressure and/or blood glucose are not under control, who have successfully completed their review at the time of the study | Numerator: Number of current beneficiaries whose blood pressure and/or blood glucose are not under control, who have successfully completed their review at the time of the study | | Review of WHV’s line list of MTM beneficiaries  Telephonic study of referred beneficiaries  Review of MTM treatment cards (whenever possible)  Review of MTM register at PHC |  |  |
|  |  |  | Denominator: Total number of beneficiaries referred by the WHV for a non-routine review over the past eight weeks | |  |  |  |
|  | 1.C.13 | Proportion of current beneficiaries of the scheme (receiving OHA for at least eight weeks) who have their Fasting Blood Sugar less than 140 mg/dL | Numerator: Number of current beneficiaries of the scheme receiving mediations for at least eight weeks) who have their Fasting Blood Sugar less than 140 mg/dL | | Review of WHV’s line list of MTM beneficiaries | Perceptions regarding their ability of patients to maintain their blood pressure and blood sugar in the context of home-delivery of drugs  Potential reasons for poor control among beneficiaries | In Depth Interviews with current beneficiaries of the scheme whose diabetes and hypertension are not under control  In Depth Interviews with WHVs |
|  |  |  | Denominator: Total number of current beneficiaries of the scheme receiving OHA for at least eight weeks | |  |  |  |
|  | 1.C.14 | Proportion of current beneficiaries of the scheme (receiving antihypertensive mediations for at least eight weeks) who have their blood pressure under control (Systolic BP <140 and Diastolic BP <90) | Numerator: Number of current beneficiaries of the scheme receiving antihypertensive mediations for at least eight weeks) who have their blood pressure under control (Systolic BP <140 and Diastolic BP <90) | | Review of WHV’s line list of MTM beneficiaries |  |  |
|  |  |  | Denominator: Total number of current beneficiaries of the scheme receiving antihypertensive mediations for at least eight weeks | |  |  |  |
|  | 1.C.15 | Proportion of current beneficiaries line listed under the HSC who are receiving medications at home | Numerator: Number of current beneficiaries line listed under the HSC who are receiving medications at home | | HSC line list of MTM beneficiaries |  |  |
|  |  |  | Denominator: Total number of current beneficiaries line listed under the HSC | |  |  |  |
|  | 1.C.16 | Proportion of current beneficiaries receiving medications at home, who are eligible for home-based drug delivery  i. Line-listed NCD patients who are 45 years and above  ii. HT/DM patients who are Home bound or have restricted mobility due to various health conditions | Numerator: Number of current beneficiaries of the scheme receiving drugs at home, who fall under the eligible category | | HSC line list of MTM beneficiaries  Review of WHV’s line list of MTM beneficiaries | Potential inclusion of ineligible population and reasons for having to do that | In Depth Interviews of WHVs and facility-based stakeholders |
|  |  |  | Denominator: Total number of current beneficiaries of the scheme compiled from the surveyed WHVs | |  |  |  |
|  | 1.C.17 | Proportion of current beneficiaries line listed under the HSC who are receiving treatment exclusively at the facility | Numerator: of current beneficiaries line listed under the HSC who are receiving treatment exclusively at the facility | | HSC line list of MTM beneficiaries |  |  |
|  |  |  | Denominator: Total number of current beneficiaries of the scheme compiled from the surveyed HSCs | |  |  |  |
|  | 1.C.18 | Proportion of current beneficiaries line listed under the HSC who are receiving palliative care services | Numerator: of current beneficiaries line listed under the HSC who are receiving palliative care services | | HSC/PHC line list of MTM beneficiaries | Perceptions regarding the performance and behaviour of palliative care nurses  Experience with the service | In Depth interviews with the beneficiaries |
|  |  |  | Denominator: Total number of current beneficiaries of the scheme compiled from the surveyed HSCs | |  |  |  |
|  | 1.C.19 | Proportion of current beneficiaries line listed under the HSC who are receiving physiotherapy services | Numerator: of current beneficiaries line listed under the HSC who are receiving physiotherapy services | | HSC/PHC line list of MTM beneficiaries | Perceptions regarding the performance and behaviour of physiotherapists  Experience with the service | In Depth interviews with the beneficiaries |
|  |  |  | Denominator: Total number of current beneficiaries of the scheme compiled from the surveyed HSCs | |  |  |  |
|  | 1.C.20 | Proportion of current beneficiaries line listed under the HSC who are receiving CAPD bags | Numerator: of current beneficiaries line listed under HSC who are receiving CAPD bags | | PSC/HSC line list of MTM beneficiaries | Experience with the service | In Depth interviews with the beneficiaries |
|  |  |  | Denominator: Total number of current beneficiaries of the scheme compiled from the surveyed HSCs | |  |  |  |
|  | 1.C.21 | Proportion of current beneficiaries who are aware of the timing for routine facility visit | Numerator: of current beneficiaries who are aware of the timing for routine facility visit (once in three months) | |  |  |  |
|  |  |  | Denominator: Total number of current beneficiaries of the scheme compiled from the surveyed WHVs | |  |  |  |

**Table 2. Program matrix to appraise the pattern of indenting of drugs across government facilities under MTM**

| **Level** | **Program element** | | **Quantitative methods** | | | **Qualitative methods** | |
| --- | --- | --- | --- | --- | --- | --- | --- |
|  | | | Indicators | Data needed | Data collection method | Data needed | Data collection method |
| Input  (2.A) | | 2.A.1 | Proportion of Oral Hypoglycemic Agents (OHA) indented at primary/secondary/tertiary health facilities between January- May, 2018 | Numerator: Quantity of OHA indented at primary/secondary/tertiary health facilities between January- May, 2018 | Analysis of drug lifting data from the district warehouses of the selected facilities between January-May 2018 | - Perceptions regarding the patterns of drug indenting and procurement at various health facilities consequent to the implementation of MTM   Challenges in streamlining patients from DME/DMS institutions to DPH institutions | In Depth Interviews with:   1. DDHS- Nodal Officer 2. District Program Officers 3. State Nodal Officers for DPH, DMS and DME   In Depth Interviews with:   1. a. DDHS- Nodal Officer 2. District Program Officers 3. State Nodal Officers for DPH, DMS and DME 4. Institutional Medical Officers |
|  |  |  |  | Denominator: Total quantity of OHA indented at all government health facilities (primary, secondary & tertiary) between January- May, 2018 |  |  |  |
|  |  | 2.A.2 | Proportion of Oral antihypertensive medications indented at primary/secondary/tertiary health facilities between January- May, 2018 | Numerator: Quantity of antihypertensive medications indented at primary/secondary/tertiary health facilities between January- May, 2018 | Analysis of drug lifting data from the district warehouses of the selected facilities between January-May 2018 |  |  |
|  |  |  |  | Denominator: Total quantity of antihypertensive medications indented at all government health facilities (primary, secondary & tertiary) between January- May, 2018 |  |  |  |
|  |  | 2.A.3 | Proportion of Oral Hypoglycemic Agents (OHA) indented at primary/secondary/tertiary health facilities between January- May, 2022 | Numerator: Quantity of OHA indented at primary/secondary/tertiary health facilities between January- May, 2022 | Analysis of drug lifting data from the district warehouses of the selected facilities between January-May 2022 |  |  |
|  |  |  |  | Denominator: Total quantity of OHA indented at all government health facilities (primary, secondary & tertiary) between January- May, 2022 |  |  |  |
|  |  | 2.A.4 | Proportion of Oral antihypertensive medications indented at primary/secondary/tertiary health facilities between January- May, 2022 | Numerator: Quantity of antihypertensive medications indented at primary/secondary/tertiary health facilities between January- May, 2022 | Analysis of drug lifting data from the district warehouses of the selected facilities between January-May 2022 |  |  |
|  |  |  |  | Denominator: Total quantity of antihypertensive medications indented at all government health facilities (primary, secondary & tertiary) between January- May, 2022 |  |  |  |

**Table 3. Program matrix to appraise the pattern of utilization across government facilities under MTM**

| **Level** | **Program element** | | **Quantitative methods** | | | **Qualitative methods** | |
| --- | --- | --- | --- | --- | --- | --- | --- |
|  | | | Indicators | Data needed | Data collection method | Data needed | Data collection method |
| Process  (3.B) | | 3.B.1 | Proportion of patients utilizing primary/secondary/tertiary health facilities for treatment of SHTN and/or DM between January- May, 2018 | Numerator: Number of patients utilizing primary/secondary/tertiary health facilities for treatment of SHTN and/or DM between January- May, 2018 | Analysis of HMIS data across primary, secondary and tertiary facilities from the selected districts between January-May 2018 | - Perceptions regarding the patterns of utilization of government health facilities consequent to the implementation of MTM - Challenges in streamlining patients from tertiary and/or secondary health facilities to primary health facilities   Perceptions and experiences of current beneficiaries who are known diabetic and/or hypertensive patients who:   1. Shifted from private facilities to government facilities for routine care after joining the scheme 2. Shifted from secondary/tertiary government facilities for routine care after joining the scheme 3. Continue to use private facilities either completely or partially for routine care after joining the scheme 4. Continue to use secondary/tertiary government either completely or partially for routine care after joining the scheme | In Depth Interviews with:   1. DDHS- Nodal Officer 2. District Program Officers 3. State Nodal Officers for DPH, DMS and DME 4. Facility-based stakeholders (Medical Officers, MTM nurses, MLHPs and/VHNs)   Qualitative case studies of current beneficiaries of MTM selected from the beneficiary list of surveyed HSCs |
|  |  |  |  | Denominator: Total number of patients utilizing all government health facilities (primary, secondary & tertiary) for treatment of SHTN and/or DM between January- May, 2018 |  |  |  |
|  |  | 3.B.2 | Proportion of patients utilizing primary/secondary/tertiary health facilities for treatment of SHTN and/or DM between January- May, 2022 | Numerator: Number of patients utilizing primary/secondary/tertiary health facilities for treatment of SHTN and/or DM between January- May, 2022 | Analysis of HMIS data across primary, secondary and tertiary facilities from the selected districts between January – May 2022 |  |  |
|  |  |  |  | Denominator: Total number of patients utilizing all government health facilities (primary, secondary & tertiary) for treatment of SHTN and/or DM between January- May, 2022 |  |  |  |
|  |  | 3.B.3 | Proportion of new beneficiaries of MTM who are known diabetic and/or hypertensive patients who routinely visited private facilities before joining the home delivery line list | Numerator: Number of new beneficiaries of MTM who were known diabetic and/or hypertensive patients who routinely visited private facilities before joining the home delivery line list | Review of WHV line list of beneficiaries  Telephonic survey of current beneficiaries |  |  |
|  |  |  |  | Denominator: Total number of known diabetic and/or hypertensive patients who newly joined the WHV’s line list over the past eight weeks |  |  |  |
|  |  | 3.B.4 | Proportion of beneficiaries of MTM who are known diabetic and/or hypertensive patients who routinely visited secondary/tertiary government facilities before joining the home delivery line list | Numerator: Number of new beneficiaries of MTM who were known diabetic and/or hypertensive patients who routinely visited secondary/tertiary government facilities before joining the home delivery line list | Review of WHV line list of beneficiaries  Telephonic survey of current beneficiaries |  |  |
|  |  |  |  | Denominator: Total number of known diabetic and/or hypertensive patients who newly joined the WHV’s line list over the past eight weeks |  |  |  |
|  |  | 3.B.5 | Proportion of beneficiaries of MTM who are known diabetic and/or hypertensive patients who continue to use private facilities for routine care either partially / completely after joining the home delivery line list | Numerator: Number of new beneficiaries who are known diabetic and/or hypertensive patients who continue to use private facilities for routine care either partially / completely after joining the home delivery line list | Review of WHV line list of beneficiaries  Telephonic survey of current beneficiaries |  |  |
|  |  |  |  | Denominator: Total number of known diabetic and/or hypertensive patients who newly joined the WHV’s line list over the past eight weeks |  |  |  |
|  |  | 3.B.6 | Proportion of beneficiaries of MTM who are known diabetic and/or hypertensive patients who continue to use secondary/tertiary government facilities for routine care either partially / completely after joining the home delivery line list | Numerator: Number of new beneficiaries who are known diabetic and/or hypertensive patients who continue to use secondary/tertiary government facilities for routine care either partially / completely after joining the home delivery line list | Review of WHV line list of beneficiaries  Telephonic survey of current beneficiaries |  |  |
|  |  |  |  | Denominator: Total number of known diabetic and/or hypertensive patients who newly joined the WHV’s line list over the past eight weeks |  |  |  |
|  |  | 3.B.7 | Proportion of beneficiaries who receive palliative care services along with the home-based delivery of medications over the past eight weeks | Numerator: Number of beneficiaries who receive palliative care services along with the home-based delivery of medications | Review of WHV line list of beneficiaries  Telephonic survey of current beneficiaries |  |  |
|  |  |  |  | Denominator: Total number of beneficiaries who newly joined the WHV’s line list over the past eight weeks |  |  |  |
|  |  | 3.B.8 | Proportion of beneficiaries who receive physiotherapy services along with the home-based delivery of medications over the past eight weeks | Numerator: Number of beneficiaries who receive physiotherapy services along with the home-based delivery of medications | Review of WHV line list of beneficiaries  Telephonic survey of current beneficiaries |  |  |
|  |  |  |  | Denominator: Total number of beneficiaries who newly joined the WHV’s line list over the past eight weeks |  |  |  |
|  |  | 3.B.9 | Proportion of beneficiaries who receive CAPD bags along with the home-based delivery of medications over the past eight weeks | Numerator: Number of beneficiaries who receive CAPD bags along with the home-based delivery of medications | Review of WHV line list of beneficiaries  Telephonic survey of current beneficiaries |  |  |
|  |  |  |  | Denominator: Total number of beneficiaries who newly joined the WHV’s line list over the past eight weeks |  |  |  |

**Table 4. Program matrix to evaluate the current status of linkage of information system under MTM with the state PHR**

| **Level** | **Program element** | **Quantitative methods** | | | | **Qualitative methods** | |
| --- | --- | --- | --- | --- | --- | --- | --- |
|  | | Indicators | Data needed | Data collection method | | Data needed | Data collection method |
| Input  (4.A) | 4.A.1 | Proportion of PHCs with computer facilities | Numerator: Number of PHCs with computer facilities/mobile/tablets | | Cross-sectional study of PHCs linked to the surveyed HSCs | Challenges faced in regular linkage of MTM beneficiary list to PHR | In Depth Interviews with:   1. MTM nurses 2. PHC Medical Officers 3. DDHS- Nodal Officer 4. District Program Officers 5. State Nodal Officers for DPH, DMS and DME 6. State-level stakeholders of MTM and PHR working with the NHM |
|  |  |  | Denominator: Total number of PHCs surveyed | |  |  |  |
|  | 4.A.2 | Proportion of PHCs/MTM nurses with access to internet connectivity | Numerator: Number of PHCs/MTM nurses with access to internet connectivity | | Cross-sectional study of PHCs linked to the surveyed HSCs |  |  |
|  |  |  | Denominator: Total number of PHCs surveyed | |  |  |  |
| Process  (5.B) | 4.B.1 | Proportion of WHV who have received training regarding linkage with PHR | Numerator: Number of WHV received training regarding linkage of MTM data with PHR | | Cross-sectional study of WHV working in the PHCs linked to the surveyed HSCs |  |  |
|  |  |  | Denominator: Total number of WHV who participated in the study | |  |  |  |
|  | 4.B.2 | Proportion of WHV who linked MTM data with PHR at least seven days over the past four weeks | Numerator: Number of WHV who linked MTM data with PHR at least seven days over the past four weeks | | Cross-sectional study of WHV working in the PHCs linked to the surveyed HSCs  Verified using Population Health registers placed at PHCs and observation of the linking process |  |  |
|  |  |  | Denominator: Total number of WHVs who participated in the study | |  |  |  |
|  | 4.B.3 | Proportion of new beneficiaries of MTM joined over the past four weeks captured under PHR | Numerator: Number of new beneficiaries of MTM joined over the past four weeks captured under PHR | | Review of HSC line list of beneficiaries  Verified using PHR database |  |  |
|  |  |  | Denominator: Total number of new beneficiaries of MTM joined over the past four weeks under the HSC | |  |  |  |
|  | 4.B.4 | Proportion of current beneficiaries of MTM under the HSC captured under PHR | Numerator: Number of current beneficiaries of MTM captured under PHR | | Review of HSC line list of beneficiaries  Verified using PHR database |  |  |
|  |  |  | Denominator: Total number of beneficiaries of MTM under the HSC | |  |  |  |
